# Dynamical Relations in the Self-Pattern

**DOI:** 10.3389/fpsyg.2018.00664

**Published:** 2018-05-11

**Authors:** Shaun Gallagher, Anya Daly

**Affiliations:** ^1^Department of Philosophy, University of Memphis, Memphis, TN, United States; ^2^Philosophy, Faculty of Law, Humanities and the Arts, University of Wollongong, Wollongong, NSW, Australia; ^3^School of Philosophy, University College Dublin, Dublin, Ireland

**Keywords:** self, pattern, self-model, predictive processing, Free Energy Principle, psychopathology, narrative

## Abstract

The notion of a self-pattern, as developed in the pattern theory of self (Gallagher, 2013), which holds that the self is best explained in terms of the kind of reality that pertains to a dynamical pattern, acknowledges the importance of neural dynamics, but also expands the account of self to extra-neural (embodied and enactive) dynamics. The pattern theory of self, however, has been criticized for failing to explicate the dynamical relations among elements of the self-pattern (e.g., Kyselo, 2014; Beni, 2016; de Haan et al., 2017); as such, it seems to be nothing more than a mere list of elements. We’ll argue that the dynamics of a self-pattern are reflected in three significant and interrelated ways that allow for investigation. First, a self-pattern is reflectively reiterated in its narrative component. Second, studies of psychiatric or neurological disorders can help us understand the precise nature of the dynamical relations in a self-pattern, and how they can fail. Third, referencing predictive processing accounts, neuroscience can also help to explicate the dynamical relations that constitute the self-pattern.

## Introduction

In contrast to the notion that a self is a mere phenomenal image produced by neural representations, as proposed by Thomas Metzinger and others, we advance a pattern theory of self as a positive account that has a number of distinct advantages. This account affirms that selves are only ever apprehended as immersed in a meaningful world; it accommodates change and adaptation to context; and at the same time it acknowledges a coherent organization as the locus of experience and self-ascription. On this account, dynamical self-patterns^1^ involve and are revealed in self-narratives, which track regenerative self-organizing processes and the various disruptions of those processes in anomalous experience. Such patterns and disruptions are also reflected in dynamical neural processes. Self, described in terms of such dynamical neural and narrative processes, is not a fixed entity but is rather an ongoing production which brings a real but contingent coherence to an evolving (or in some cases, devolving) stream of sensations, thoughts, emotions, desires, memories, and anticipations.

Although Metzinger’s (2004) notion of a self-model indicates the relevance of brain processes and their dynamical connections for an understanding of the concept of self, he also contentiously proposes that “[n]obody ever *was* or *had* a self” (p. 1). To be clear, with this assertion Metzinger is rejecting accounts that explicitly endorse or implicitly underwrite substantively “real” Cartesian selves, i.e., dualistic conceptions of selves as grounded in separate substances. We do not dispute this. There are, however, some challenges that Metzinger’s account needs to address. First, if a self is not real the way a substance is real, it is also the case that the self is not non-real in the way an illusion is non-real(Metzinger, 2004, p. 209). Accordingly, one needs to specify in what way we may talk about the reality of the self.

Second, according to Metzinger, the phenomenal self is generated in a process involving neuro-dynamics, from which subjective self-awareness emerges. The key challenge for this approach is to explain precisely how a self-model is related to or generated in the brain. As we’ll show in section “Neural Patterns and the Self,” there seems to be no agreement about what brain processes are relevant to a self-representational system, or whether there are specialized or exclusive self-specific areas in the brain. Alternatively, one could appeal to predictive processing models. Metzinger’s (2004) original proposal was already sketching such a model (pp. 51–52). More recently Metzinger has moved further in that direction (Metzinger, 2013, 2014; Wiese and Metzinger, 2017), endorsing the idea of an automatic and continuing minimization of prediction error by which the brain monitors the body and immediate environment for potential unexpected events (citing, e.g., Friston, 2005; Hohwy, 2013; Limanowski and Blankenburg, 2013; Seth, 2013).

An alternative way to think of a self-model is to consider it as irreducible to brain processes, although brain processes are not irrelevant to explaining self-model integration. Self-models, we will argue, additionally depend on a variety of extra-neural factors, such as bodily processes and processes that are social and cultural, and are generated in the organism’s relations with its environment. This is the concept of a pattern theory of self (Gallagher, 2013). On this view, the self has the scientifically useful reality of a pattern (Dennett, 1991). One challenge associated with this view is to demonstrate the dynamical relations amongst elements that constitute a self-pattern. We wish to emphasize the point that while the brain is not the sole generator of the self-pattern, mapping of self-related brain function may help to demonstrate the dynamical and relatively coherent nature of self-patterns. Is it possible, in this respect, that some version of the predictive processing model might provide a means to clarify these dynamical relations?

In this paper, after reviewing some issues concerning self and the brain, we’ll pursue the alternative concept of a pattern theory of self. We’ll answer some objections that have been raised concerning the dynamical relations amongst different elements of a self-pattern. We’ll argue that there are three ways to track the dynamical relations in a self-pattern: first, through narrative; second, through the study of psychopathology; and third, through predictive processing in the brain^2^.

## Neural Patterns and the Self

A number of approaches have attempted to relate neural processing to the concept of self. Karl Popper and John Eccles, for example, defended a dualist view that treated the self as an autonomous entity that interacted with neural processes, and purportedly controlled them. “The self-conscious mind acts upon … neural centres, modifying the dynamic spatio-temporal patterns of the neural events” (Popper and Eccles, 1977, p. 495; Eccles, 1989, 1994). This non-reductionist, Cartesian view, however, did not represent either the philosophical or neuroscientific consensus of the time, and still does not. It did motivate a debate between the philosopher Mario Bunge, who defended an emergentist view, and the neuroscientist Donald MacKay, who, although starting “from our immediate experience of what it is like to be a person” (MacKay, 1978, p. 601), staked out a position between materialism and dualism (Bunge, 1977, 1979; MacKay, 1978, 1979, 1980). MacKay argued that since each change in experience corresponds to a change in brain activity, there is both first-person data and third-person data about the conscious self. Importantly, for MacKay, despite the high degree of correlation between these data sets, one set is not reducible to the other set.

In these various discussions, the concept of self was never clearly defined. Since the 1970s, however, there have been both clarifications and complications introduced around the notion of self as it is discussed in both philosophy and neuroscience, and there have been important qualifications made regarding correlations between brain and self. For example, LeDoux (2002, p. 31) has pointed out that theories of the self “are not usually framed in ways that are compatible with our understanding of brain function.” This idea is echoed by Northoff et al. (2006, p. 453): “Neither the historical nor modern psychological approaches are obviously isomorphic with any known brain analysis.” The lack of a perfect correlation between self and the brain thus results in worries such as those expressed by Apps and Tsakiris (2014, p. 85): “recent reviews of this literature have concluded that the absence of a unifying theoretical framework has resulted in a largely incoherent picture of the circuits and mechanisms which are engaged during self-recognition.”

Such thoughts have not stopped the continuing analysis of how various aspects of self relate to brain, however. Much of this research asks how specific brain areas correlate with self-related phenomena. Thus, for example, autobiographical knowledge, personal beliefs, self-conceptions, and the recognition of our own face have been grouped together as related to left hemisphere activity (Turk et al., 2003; see also Kircher et al., 2000). At the same time there is evidence that “processing of self-related information (e.g., autobiographical memory, self face identification, theory of mind) is related to activity in the right frontal cortex” (Platek et al., 2003, p. 147; see also Devinsky, 2000; Miller et al., 2001). Representations of both the physical and phenomenological aspects of self, and “self-representation in general” is said to involve the right lateral parietal cortex (Lou et al., 2004, p. 6831), while the “self model … a theoretical construct comprising essential features such as feelings of continuity and unity, experience of agency, and body-centered perspective” (Fossati et al., 2003, p. 1943) involves activation of the medial prefrontal cortex in both hemispheres.

Cortical midline structures (CMS) have been shown to process information related to self. In particular, activation of the ventromedial prefrontal cortex (VMPFC) has been repeatedly observed when subjects think about themselves (Northoff and Bermpohl, 2004; Northoff et al., 2006) and when they make judgments about their own personalities (Johnson et al., 2002; Kelley et al., 2002; Heatherton et al., 2006; D’Argembeau et al., 2007; Gutchess et al., 2007; Ruby et al., 2009). Lesion studies also show that damage to the VMPFC leads to deficits in self-awareness (Stuss et al., 2001a,b). Northoff thus argues for a unitary neural network or system responsible for all self-related phenomena (Northoff and Bermpohl, 2004; Northoff et al., 2006). The putative CMS self-system, however, covers a quite large set of brain regions (including ventro- and dorsomedial prefrontal, anterior and posterior cingulate, retrosplenial, and medial parietal cortices). It involves a multitude of connections between CMS and subcortical areas, and the possible role of subcortical areas related to an embodied self (Northoff and Panksepp, 2008).

The large number of cortical areas that correlate with self-reference or self-experience suggests that there is no specialized brain area *simpliciter* responsible for generating “the self” (Gillihan and Farah, 2005). LeDoux (2002) notes the complexity: “different components of the self reflect the operation of different brain systems, which can be but are not always in sync” (p. 31). It is also likely that no area of the brain is exclusively self-specific (Legrand and Ruby, 2009). According to Craik et al. (1999, p. 30), “every significant activation in the [self condition] was also found in either the [other person condition] or the [general semantic] condition, or both” (also Gillihan and Farah, 2005, p. 94). Moreover, most of the cited studies focus on self-referential tasks, i.e., tasks that take the self or self-aspect to be part of the object of consciousness – e.g., thinking about myself, judging my personality traits, self-face identification. As Legrand and Ruby (2009) make clear, this focus misses pre-reflective self-awareness, in which I am not aware of the self as an object, but am self-aware in a non-observational way, as the experiencing subject. The notion of ecological self-awareness (Neisser, 1988) is an example of this kind of pre-reflective self-awareness. The fact that I always perceive from a first-person (egocentric) perspective suggests that there is always a proprioceptive sense of this self-related perspective implicit in my experience, sometimes called the sense of mineness or the sense of ownership. Metzinger’s self-model also includes elements related to first-person perspective and body schematic processes that are associated with pre-reflective bodily processes (Blanke and Metzinger, 2009; Limanowski and Blankenburg, 2013).

Rather than finding a common, well-circumscribed brain area of self-referential activation, then, these various studies indicate a large number of diverse areas activated for a variety of self-related experience, but with no area ever activated exclusively for self. This led Vogeley and Gallagher (2011) to conclude that the self is both everywhere and nowhere in the brain, and to offer a caution.

To achieve clarity in neuroscientific studies of self, it is incumbent on researchers to define the precise aspect of self under study. Selves are experiential, ecological, and agentive; they are often engaged in reflective evaluations and judgments; they are capable of various forms of self-recognition, self-related cognition, self-narrative, and self-specific perception and movement. In many of these activities, selves are more “in-the-world” than “in-the-brain,” and they are in-the-world as-subject more so than as-object. (Vogeley and Gallagher, 2011, p. 129).

In summary, explaining precisely how a self-model is related to or generated by neuronal processes remains a key challenge for any account of self. There is, as yet, no agreement about what brain processes are relevant to a self-representational system, or whether there are specialized or exclusive self-specific areas in the brain.

## A Pattern Theory of Self (PTS)

The pattern theory of self (PTS) attempts to capture both the plurality of factors involved in self and the idea that the self (as an agent) is more “in-the-world” than “in the brain”^3^. In brief, PTS argues that a self is constituted as a pattern of a sufficient number of characteristic factors, including embodied, experiential, affective, behavioral, intersubjective, psychological/cognitive, reflective, narrative, extended, and normative factors (see **Table 1**). Importantly, this is not intended to be an additive list of factors, but a set of components dynamically interrelated in a pattern or gestalt arrangement (Gallagher, 2013). This means that an intervention that affects one factor will involve modulations in the other factors. Adjustments in one aspect, above a certain threshold, will lead via dynamical interactions to changes in others. For example, very basic aspects of self-experience, such as the sense of agency, can be modulated by more complex, relational aspects, such as social normative factors that involve culture, gender, race, health, etc., and by specific intersubjective factors that can either diminish or enhance one’s autonomy and sense of agency (Young, 1980; Gatens, 1996; Weiss, 1999; Murphy A., 2008; Gallagher, 2012; Leach-Scully, 2012; Carel, 2016).

**Table 1:** Dynamical aspects of the self-pattern.

Elements of the pattern	Brief description
Embodied elements	Core biological, ecological and interoceptive factors, allowing the system to distinguish between itself and what is not itself – extremely basic to all kinds of animal behavior.
Minimal experiential elements	First-person, pre-reflective, conscious experience, reflecting the self/non-self distinction, manifest in various sensory-motor modalities (kinesthesia, proprioception, touch, vision, etc.) – including a *sense of ownership* (the “mineness” of one’s experience) and a *sense of agency* for one’s actions (Rochat, 2011; Gallagher, 2012).
Affective aspects	Affect/emotion/temperament, ranging from bodily affects to what may be a typical affective or emotion pattern(Newen et al., 2015).
Behavioral aspects	Behaviors and actions make us who we are – behavioral habits reflect, and perhaps actually constitute, our character. This is a classic view that goes back at least to Aristotle.
Intersubjective interactions and capacities	Human are born with a capacity for attuning to inter-subjective existence, which develops into a social self-consciousness – a self-for-others (Mead, 1913), manifested behaviorally in mirror self-recognition (Gallup et al., 2011), and the neuronal mirror system (Gallese, 2014).
Psychological/cognitive elements	Traditional theories of the self focus on these factors, which may range from explicit self-consciousness to a conceptual understanding of self as self, to personality traits of which one may not be self-conscious at all – psychological continuity and the importance of memory are highlighted in the literature on personal identity (Shoemaker, 2011).
Reflective capacities	The ability to reflect on one’s experiences and actions – closely related to the notions of autonomy and moral personhood, including the capacity to reflect and form second-order volitions about one’s desires (Frankfurt, 1982; Taylor, 1989).
Narrative capacities	Although some theorists make the strong claim that narratives are constitutive for selves (Schechtman, 2007, 2011), for PTS one can lose the ability to construct a self-narrative (as in cases of dysnarrativa) and still remain a self to the extent that other elements of the pattern remain in place.
Extended/situated elements	Including the possibilities presented by physical pieces of property, and various things that we own (James, 1890). Not only may we identify with our material belongings, the technologies we use, our professions, and the institutions we work in, but we are dynamically related to the action possibilities they afford.
Normative factors	Ranging across possibilities presented by the kind of family structure and situation in which we grew up to cultural and normative practices, involving physical and mental health, gender, race, and economic status, that define our way of living.


According to PTS, selves are individuated as patterns of characteristic features, no one of which is sufficient for the existence of a particular self. Specifications of which features contribute to a self-pattern, and how they may be arranged (e.g., whether some have primacy, or whether there might be hierarchical arrangements among factors) are open to contentious debate among philosophers and cognitive scientists who defend opposing theories. Rather than deciding this issue *a priori*, PTS treats it as an empirical question that may be answered differently in different cases. It does not stipulate in advance what features need to be present in each instance.

Although we emphasize the dynamical nature of the self-pattern, such that particular elements or factors are to be considered dynamically interwoven with other elements, one important criticism of PTS has been that there is so far no explanation of these dynamical relations. Thus, for example, Miriam Kyselo (2014) writes: “Once the diversity of self related phenomena is acknowledged [as in PTS], we also need to understand how the elements of a collection of relevant self features interrelate. A pattern approach to the self acknowledges diversity but lacks integration, offering no account of the individual as explanatory whole” (p. 1; also see Beni, 2016). Likewise, de Haan et al. (2017) suggest that the main weakness of PTS is that “it is just a list; a heaping of aspects, without an account of how they relate. There are no considerations on their potential ordering, hierarchy or structure. But in the case of psychiatric disorders and their treatment the relevant questions precisely pertain to this structure, to the relation between aspects of the self” (pp. 5–6).

Both Kyselo and de Haan are concerned about how PTS might explain various pathologies. As Kyselo rightly suggests, researchers in cognitive science as they set up experiments, and psychiatrists as they evaluate patients and attempt to understand pathologies of the self, need to have a conception of the self as an explanatory whole. PTS is nonetheless consistent with Kyselo’s suggestion that an embodied-enactivist approach may be able to offer an integrative perspective on the self as a whole. Yet we cannot determine the nature of the dynamical links among the various factors *a priori*, or simply by adopting a particular theory. We read Kyselo’s proposal as suggesting that we need to look in the direction of empirical examples, including cases of psychiatric or neurological disorders, to sort out the precise nature of the dynamical relations that would help us to understand how selves can be integrated or disintegrated in particular cases. Rather than an alternative to PTS, as Kyselo conceives it, however, working out the dynamical relations would provide a more detailed account of PTS.

From the perspective of PTS, it is not the case that one needs to add something extra to the specific pattern dynamically formed by the interrelations of the various elements in order to be able to identify the coherency or structure or ordering behind the diversity; rather the coherency, structure or ordering will be reflected in the pattern that emerges from these interrelations. Dynamical connections of the pattern themselves get reflected in three significant and interrelated ways that allow for investigation. First, a self-pattern is reflectively reiterated in its narrative component. That is, self-narratives reiterate the other components and the dynamical relations of a self-pattern in a way that will allow us to make sense of these relations (see section “What’s the Use of Narrative?”). Second, as already indicated, the dynamics of a self-pattern are revealed in the way that they break down in pathology. Empirical, clinical, and phenomenological studies of psychiatric or neurological disorders can sort out the precise nature of the dynamical relations in a self-pattern (see section “Psychopathology”). Third, neuroscience can also help to explicate the dynamical relations that constitute the self-pattern. In taking the notion of a self-pattern to be the basis of a non-reductive approach to understanding self,the intent is not to exclude neuroscience, or to downplay the importance of brain function, which underpins many of the bodily, experiential, and cognitive factors that make up a self-pattern. Indeed, as Fingelkurts and Fingelkurts (2017) suggest, changes in neurophysiology can “index” changes in the self-pattern, complementary to the way that narrative reflects such changes. In this respect, however, given the issues raised in section “Neural Patterns and the Self,” it will be more productive to look not at specific brain areas or neural representations to find correlations but more generally at dynamical processing and brain function. In section “Neuronal Patterns, Predictive Processing and Self-Patterns,” we consider the development of recent predictive processing models as a better approach to this goal.

## What’s the Use of Narrative?

One way to understand the narrative aspect of self is to take it as a window that opens onto the self-pattern and offers a way to map the dynamical relations among the various other factors of the self. Self-narratives in some sense reflect, explicitly in content, or implicitly in form, all of the other aspects of the self-pattern (Hutto and Gallagher, 2017). Specifically, narrative is a means of retrieving, disclosing, temporally mapping, and connecting all the other aspects.

To show how this works, we’ll focus on one example that we take from Frith and Metzinger (2016). They discuss the case of regret, which, as they propose, involves a form of self-knowledge. This kind of self-knowledge takes a narrative form in which I, as the narrator, can give an account, and evaluate my past action. Assuming that I have made the wrong choice sometime in the past, I come to regret that choice, and this is something that manifests itself in my self-narrative. What get reflected in my narrative in this respect are changes or adjustments in a variety of aspects of my self-pattern.

For example, the minimal experiential aspects of the self-pattern include the sense of agency and the sense of ownership. Frith and Metzinger point out, first, that the situation captured by the narrative involves a changing status of the sense of agency. I had a sense of control over my actions when in fact I made the wrong choice and acted on it. This sense of agency does not apply to my regret, however, or at least I seem unable to avoid my regret about that action. Adjustments in my sense of agency are reflected in my narrative when I say to myself or to someone else that I regret my action. In contrast, the sense of ownership pertains to both my action and my regret: “while the sense of agency is represented as something we possessed in the past, the state of regret itself does not itself involve a sense of agency. While the phenomenology of ownership is crisp and distinct (I identify with my regret, it is an integral part of myself), regret itself is not an action. It is a kind of inner pain that simply appears in us” (Frith and Metzinger, 2016, p. 202). As they also make clear, my regret is not just a characteristic that I can narrate, it is something that takes over my narration. “The phenomenology of regret can be described as a loss of control over our personal narrative, and in this sense it is also a threat to our integrity. It is a threat to the integrity of our autobiographical self-model, because, on the personal level of description, we become aware of an irrevocable damage to our life narrative” (Frith and Metzinger, 2016). The emergence of an inner pain reflects the affective aspect of the self-pattern. “Because it is an emotional and frequently also an embodied experience, perhaps with heart wrenching qualities, it is not something we can distance ourselves from – another important way in which regret involves a loss of control” (Frith and Metzinger, 2016).

The narrative is doing some work in tying together some of the other factors in the self-pattern, and forming a “transtemporal identity” of the subject across time. The relation between action and regret, despite variations in my sense of agency, means that I am the same person who acted in the past, and continues to be responsible for my actions. Frith and Metzinger suggest that this is a fictional unity: “a transtemporal, fictional ‘self”’ constituted by the narrative (Frith and Metzinger, 2016). Yet the action and affective states – the pain and the regret – are real, and are factors that specify my self-pattern.

We can also acknowledge how an intersubjective or social dimension relates to both agency and affect, since, as the narrative may reveal, others are affected (for example, frustrated) by my actions. How I relate to those others partially shapes who I am. In Frith and Metzinger’s (2016) terms, the “important point is that, for any organism that has acquired the capacity to feel regret and whose behaviour is determined by this very special form of conscious content, the self-model and the group-model have become functionally integrated in a much stronger way” (p. 204). The narrative may in fact reflect my identification with the values and desires of others, which may increase my emotional suffering “from a self-caused frustration of group-preferences” (Frith and Metzinger, 2016). I feel regret because, as one might say, my conscience has been formed by my upbringing. As they put it, the “group-model has invaded the organism’s self-model to such a degree that the conflict between group and individual interests is now internally modelled in a way that a) includes sanctions by the group (regret is internal self-sanctioning), and b) the dynamic competition between group and individual interests is now taking place not only on the level of overt, bodily actions, but has found a new platform – the self-model of the individual” (Frith and Metzinger, 2016, pp. 204–205). That is, there are dynamical adjustments in the self-pattern in which intersubjective/social factors determine my reflective evaluation and my feelings about what I have done. Most likely, these also manifest in my behavior and get reflected again (and perhaps repetitively) in my narrative.

My regret and how I, and others, evaluate my past action can have an effect on my future choice of action. My deliberations and actions relate to normative factors, to “cultural practices, such as moral codes and laws. By generating beliefs about self-control [my narrative] shapes the sense of self and gives rise to concepts like responsibility, intentionality, accountability, culpability, and mitigating circumstances” (Frith and Metzinger, 2016, p. 205). Frith and Metzinger are describing a loop through normative factors that feeds back to influence the behavior of the person, and they suggest that “the sense of agency and the idea of voluntary action are acquired through cultural learning. The causal link between the group level and the individual level is constituted by the conscious self-model, in which group preferences are increasingly reflected as social complexity increases” (Frith and Metzinger, 2016, p. 205). Alternatively, as they point out, further physical and affective changes may lead to anxiety and depression (Roese et al., 2009), delayed sleep onset and insomnia (Schmidt and Van der Linden, 2013), and onward to additional changes in self-narrative. As adjustments occur in the self-pattern, they get reflected in the self-narrative.

The point is that we can move forward on understanding the dynamical relations (between experiential, embodied, affective, social and normative factors) that constitute a coherent self-pattern as they get reflected in self-narrative. We can map out these relations by looking at instances where some action, event, or intervention causes changes in various factors in the self-pattern that get traced out in self-narrative, including those instances where the pattern becomes disordered, or ordered differently^4^.

## Psychopathology

If we avoid doctrinaire approaches to psychiatric classifications that insist on exclusive taxonomies of either natural kinds or social constructs, and opt instead for what Zachar (2003) calls “practical kinds,” it may be useful to consider the idea that all psychiatric disorders are disorders of self (Daly and Gallagher, unpublished). Psychiatric disorders tend to be multifactorial. To the extent that we think of such disorders as self-disorders, each individual case of a particular disorder may show different patterns of dynamical relations among the various factors that constitute a particular self. This is because different factors may involve different weights in different individuals. For example, anorexia will present differently in a disciplined person with high degrees of self-control than in an impulsive individual (Zachar, 2008, p. 334). Variations in the amount of social support or different cultural contexts may determine how a disorder develops. But we can expect to see typical patterns of practical kinds associated with different disorders. Schizophrenia, for example, may affect multiple aspects of experience, cognition, mood, and agency in certain typical ways, while panic disorder will be more aspect limited in its impact (Murphy D.P., 2008).

The list of elements or contributories to the self-pattern, and the dynamical relations among them can be used to specify most if not all aspects relevant to symptomology in psychiatric cases. Seeing how such aspects of the pattern undergo typical changes, comparatively in different disorders, can help to reveal the particular dynamical relations among those aspects. Here again, looking specifically at the narrative aspect can help. We can learn about pathological changes or transformations of self-patterns from what the patient reports, i.e., from her own self-narrative. For example, she may report (or her reports may reflect) the loss of a sense of agency, as in some schizophrenic symptoms, or in addiction, without losing other basic experiential elements that are part of a self-pattern. These are not uniform reports across different disorders. In the case of schizophrenic thought insertion, for example, the experience is of some other, external agent invading one’s stream of thought. In the case of obsessive compulsive disorder (OCD), the patient knows that she makes herself do what she does, but nonetheless cannot resist. In drug addiction, there are instances where there is a conflict between first-order desires and second-order, reflective intentions, where the drug wins out in opposition to what the subject really wants. In some episodes of bulimia the agent is in prospective control as she plans out and executes the binge, perhaps loses control in the middle of it, and then retrospectively feels that she was completely out of control (see Kendler, 2008 for these examples). Differences in how the sense of agency works in these different disorders impinge differently on affective aspects of the self-pattern. Addiction and bulimia may involve positive hedonic experiences at first; but this is not the case in OCD where the subject experiences no pleasure in what she is doing, and is motivated by a type of dissatisfaction or sense of incompleteness.

In other disorders, patients may exhibit loss of certain psychological and cognitive abilities, such as the ability to recall earlier events (as in amnesia or Alzheimer’s Disease). They, or their caregivers, may report these changes; or these changes may start to dramatically shape the patients’ narratives. Along with this, they may have undergone character or personality changes. In many cases, pathological changes can interfere with the individual’s autonomy and positive social engagement. In other cases, some degree of self-identity and autonomy may continue to be supported by intersubjective relations and/or extended aspects in the subject’s surroundings. In the case of depression, the patient may reveal thinking that reflects disorders in mood and affective processing as well as disrupted processes connected to the sense of agency and identity. Depressed patients can experience self-alterations and identity changes, and they will attempt to integrate these changes into their life by trying to understand the reasons they’re feeling this way. They may report feelings of helplessness, desperation, self-loathing, as well as the incapacity to work, to act, to think clearly (Solomon, 2001; O’Brien, 2004).

Mapping the changes across different aspects of self-pattern should reveal a set of underlying dynamics that get disrupted or adjusted as one change impinges on the whole pattern. That is, if the pattern is in fact a dynamical gestalt, then we should expect to find not just isolated symptoms affecting one aspect alone, but readjustments (disruptions or compensatory adjustments) in some set of other aspects. One tends to see this in light of multiple factor theories of different disorders. If, for example, in schizophrenia, one’s minimal experience of the sense of agency is disrupted (due to some neurological malfunction), this can lead to cognitive-based delusions, which in turn can impinge on affect and social relations, as well as changes in the structure of the patient’s narratives and self-concept (Gallagher, 2000, 2003; Frith and Gallagher, 2002; Berna et al., 2016).

In the case of psychopathology, self-narratives can provide a forensic measure, a linguistic fingerprint, of different conditions (Gallagher and Cole, 2011). Studies of the narratives of people with psychopathology show different sets of dynamical relations among aspects of self-pattern. Junghaenel et al. (2008) found a lower frequency of cognitive words (e.g., cause, know, ought) in the narratives of psychiatric patients in general, signaling some kind of cognitive change. In various depression narratives, alterations in what Ratcliffe (2014) calls existential feelings can be mapped out across different components of the self-pattern, including bodily and experiential changes (Fuchs, 2005; Slaby and Stephan, 2008; Fuchs and Schlimme, 2009; Paskaleva, 2011). Narrative structures themselves, including temporal and syntactical structures, are disrupted in depression. Pennebaker et al. (2003) show that increased use of the first-person pronoun in self-narratives predicts both depression and mania. Stirman and Pennebaker (2001), in a study of artists who committed suicide compared to those who did not, found relatively higher frequencies of self-reference words (e.g., I, me, my) as compared to other-reference words (e.g., we, them, they). Likewise, in schizophrenia self-narratives can reflect the disruption of the dynamics of the self-pattern (Bovet and Parnas, 1993; Gallagher, 2003; Phillips, 2003). Gruber and Kring (2008) showed that for schizophrenics, negative emotion narratives were less grammatically clear than positive ones, and positive emotion narratives were more likely to involve other people than negative narratives. It’s also the case that schizophrenic patients who narrate emotionally loaded facts (vs. neutral facts) showed higher degree of dyslogia or wrong or novel words (Caixeta et al., 1999). In schizophrenia, self-narratives are less coherent and elaborate than controls, suggesting problems connecting self and memory (Raffard et al., 2010; Berna et al., 2016). Lysaker et al. (2002, 2005) looking at narrative, metacognition, and self-esteem, developed a helpful tool, the Scale to Assess Narrative Development (STAND), which can capture some of these changes.

In the case of Major Depressive Disorder (MDD), almost all factors in the self-pattern may be affected. The following list is based on Daly and Gallagher (unpublished). Those aspects listed under (a) are derived from the DSM-V list of symptoms. Those listed under (b) are from a variety of interviews, vignettes, and patient narratives collated from various sources ^5^.

• In regard to *embodied elements*, the subject may undergo (a) significant changes in weight, insomnia or hypersomnia; (b) lack of appetite, slowness of movement.
• *Experiential aspects* may involve (a) fatigue or loss of energy; (b) feelings of heaviness, feeling disembodied or hyperembodied, feeling like an automaton (reduced sense of agency).
• *Affective aspects* may include (a) depressed mood (sadness, emptiness, hopelessness), diminished interest, feelings of worthlessness or guilt; (b) loss of empathic resonance with others, loneliness, self-loathing or low self-esteem, pervasive sense of dread, unaccountable fears, feeling that one’s experience is absolutely private and absolutely isolating, despair, feeling of being excluded, not understood, underappreciated, self-alienation.
• *Behavioral aspects* may include (a) psychomotor agitation or retardation (observable by others); (b) inability to stop crying, diminished physical self-care, self-harm to reduce anxiety.
• *Intersubjective aspects* may involve (a) … (b) feeling like a burden for others, like a loser, feeling excluded – feeling of not belonging, profound intersubjective alienation, concern that others think they are malingerers, negative assessment of self-appearance (in mirror), feeling invisible.
• *Psychological/cognitive aspects* may include (a) diminished ability to think or concentrate, indecisiveness, recurrent thoughts of death, recurrent suicidal ideation without a specific plan, or formulation of a specific plan for suicide; (b) disordered attention, excessive rumination, toxic thought processes, mind fog, nihilism, difficulty in imagining a different future, changed time perception, time experienced as passing very slowly, closure of the future.
• *Narrative aspects* may include (a) … (b) repeatedly re-scripting conversations that were deemed unsatisfactory, predominance of use of first person pronouns, past narratives are couched in terms of loss, failure, and damage, present narratives hold little or no interest, future narratives have dried up.
• *Extended/situated aspects* may include (a) … (b) things and surroundings experienced as less salient; diminished engagement with the world; loss of sense of belonging or fitting in place.

To define the dynamical connections among these various changes one might have to track the etiology and temporal development of specific symptoms and/or recovery during treatment. We can expect that in recovery, some symptoms may respond quickly while others lag behind or may require different treatments.

## Neuronal Patterns, Predictive Processing, and Self-Patterns

Studies of depression suggest that in the depressed subject, abnormal dynamical synchronies exist between the various factors of first-person perspective, bodily/emotional agency, and reflective (narrative related) agency, as measured by dynamical connections across correlated brain areas (Fingelkurts and Fingelkurts, 2017). Patients with MDD “have abnormal self-related processing, mostly expressed as increased self-focus, excessive self-reflection (rumination) and association of the self with negative emotions…. Generally, excessive ruminative self-focus produces such feelings as worry, guilt, shame, jealousy, which may lead to insomnia … increased anxiety…. Patients with depression showed a higher degree of interoceptive awareness [… and] distorted body self-image….” (Fingelkurts and Fingelkurts, 2017, p. 30). As these researchers suggest, such aspects of selfhood “are not entities that simply modify something that has its own independent existence, but rather together form a dynamic pattern, that as a whole constitutes a complex selfhood” (Fingelkurts and Fingelkurts, 2017, p. 35). The hypothesis that the dynamical relations among these aspects may be reflected in dynamical neural connections offers another way to make such relations open to investigation^6^.

This hypothesis is defended within the framework of predictive processing and *the free energy principle* (FEP) in several recent papers^7^. Limanowski and Blankenburg (2013), for example, argue that the minimal (pre-reflective) experiential aspects of the bodily self, including the first-person perspective, and the senses of agency and ownership or mineness, all of which depend on multisensory integration, including interoception, can be “mapped onto a hierarchical generative model furnished by the FEP and may constitute the basis for higher-level, cognitive forms of self-referral” (p. 1; also see Fotopoulou, 2012). Beyond minimal experiential aspects of the self, multisensory integration on the predictive processing model can also explicate connections to affective factors (Seth, 2013), and solve problems pertaining to self-recognition, which may involve recognition of the self-as-object, as in, for example, mirror self-recognition (Apps and Tsakiris, 2014). As Apps and Tsakiris point out, this approach can explain how self-specific neural processing may arise in any multisensory processing, without positing specialized circuits or parts of the brain that are self-specific (important for avoiding the problems outlined in section “Neural Patterns and the Self”). Indeed, predictive processing accounts suggest that although self-recognition may involve the specific congruency of predictions driven by efference processes and incoming sensory input (e.g., Frith, 1992; Gallagher, 2000; Friston et al., 2010; Brown et al., 2013), the FEP framework “provides flexibility, with fewer constraints on what types of information can drive self-recognition” (Apps and Tsakiris, 2014, p. 89). In this respect, the claim is that predictive models provide the resources to explain all of the various factors that contribute to the self-pattern. As Apps and Tsakiris (2014) note, “This is particularly important, given the evidence to suggest that the continuity of the self may be underpinned by many different types of information, the integration of which leads to a coherent sense of one’s body” (p. 90^8^).

Predictive models conceive of a system such as the brain or the organism as perceiving or coping with the world by predicting what it will encounter, and then minimizing prediction errors generated in processing sensory input. The uncertainty of experience is measured in terms of the quantity of prediction errors (free energy) in the system. The system reduces free energy by revising its predictions or by taking action to change its environment to match its predictions, or both. Although internalist descriptions of predictive processing conceive of the brain as hypothesizing (or becoming) a “hierarchical generative model” of the world (Hohwy, 2013), other descriptions emphasize active, embodied engagement (“active inference”) with the aim of maintaining an organism-environment attunement (Bruineberg et al., 2016; Gallagher and Allen, 2016; Clark, 2017; also see Hohwy and Michael, 2017 on this point).

The minimal experiential elements of the self-pattern are based on a model that “successfully predict[s] or match[es] the sensory consequences of our own movement, our intentions in action, and our sensory input” (Limanowski and Blankenburg, 2013, p. 3; citing Hohwy, 2007, p. 9). “In short, the notion that there is a ‘self’ is the most parsimonious and accurate explanation for sensory inputs. In mathematical terms, this parsimonious accuracy is exactly the quantity that is optimised when minimising free energy or prediction error” (Apps and Tsakiris, 2014, p. 89; Hohwy and Michael, 2017).

The embodied system predicts and integrates both exteroceptive and interoceptive multisensory variations in a probabilistic way as we perceive and move and experience ourselves moving. The integrated sensory experience is not *about* the self (in the sense of taking the self as an intentional object); it’s about the world and at the same time (in an ecological fashion) it registers bodily states and self-movement. This cross-modal, self-correcting interoceptive, proprioceptive, efferent/afferent and exteroceptive integration generates a self-model (Metzinger, 2004), manifested phenomenologically in a body-centered spatial frame of reference.

Following this logic, higher-level multisensory areas must predict input in multiple sensory modalities, which according to Apps and Tsakiris (2014) implies “a high level representation [or model] (of self) that elaborates descending predictions to multiple unimodal systems” (Limanowski and Blankenburg, 2013, p. 3)^9^.

As Apps and Tsakiris (2014) and Limanowski and Blankenburg (2013) agree, this self-model is plastic so that basic factors such as the experience of self-location (location of one’s body) and the sense of ownership can be manipulated through experimental variances in stimulation such as in the rubber hand illusion (RHI) or whole-body displacement (Botvinick and Cohen, 1998; Lenggenhager et al., 2007; Blanke, 2012). These minimal experiential aspects of the self-pattern result from the self-specifying integration of bottom-up input (e.g., the combination of visual and tactile input in the case of the RHI) and top-down factors or priors (e.g., the habitual predictions associated with body image), which drives the illusion of ownership of the rubber body part. The illusory model (which generates the experience of the felt and the observed touch as at the same location) “is selected [by the brain] because it more successfully explains the incoming prediction error in favor of a unified self” (Limanowski and Blankenburg, 2013; see Hohwy, 2013). This model minimizes prediction error (free energy); it can be further refined toward a more veridical model by active inference, i.e., by moving my hand. Getting a veridical model of my body and its relation to the environment, or something close to a veridical model (reducing free energy in the system), is important for survival; the self exists “iff (sic) [sic] I am a veridical model of my environment” (Limanowski and Blankenburg, 2013, p. 4; quoting Friston, 2011).

This last thought, that the organism (the bodily self), rather than “having” a model, *is* itself the model of the environment is, as we understand it, Friston’s way of emphasizing the strong connectivity between organism and environment – who we are, even in a very basic embodied way, is shaped by the environment, our interactions with, and our actions in the environment. In terms of the PTS, this suggests a dynamical integration between the embodied and experiential aspects of the self-pattern and the extended and normative aspects (understanding the environment to be social/cultural as well as physical) ^10^. Evidence for the close connection (which could be modeled as a dynamical causal reciprocity) between embodied (interoceptive) factors and experiential aspects can also be found in (1) the fact that during the rubber hand illusion the temperature of one’s real hand drops as the sense of ownership shifts to the rubber hand (Moseley et al., 2008). This is “interpreted as evidence for top-down regulations of autonomic control and interoceptive prediction error minimization during the RHI” (Limanowski and Blankenburg, 2013, p. 4); and (2) the idea that self-awareness emerges “from interaction of interoceptive predictions and prediction errors” (Limanowski and Blankenburg, 2013, p. 5, citing Critchley and Seth, 2012). Furthermore, Seth (2013); Seth et al. (2011), and Suzuki et al. (2013) suggest that interoceptive (e.g., autonomic) and efferent or motor control signals, operating as part of the self-model (or according to PTS, the embodied factors of the self-pattern), give rise to “emotional feeling states.”

Limanowski and Blankenburg (2013) provide further analysis from this perspective to explain how the sense of agency and sense of mineness emerge as part of the experiential aspect of the self-pattern, and how predictive processing also helps to explicate intersubjective self–other relations. Here, then, we see ways to connect embodied, minimal experiential, affective, and intersubjective aspects of the self-pattern. The point we want to highlight here is that predictive processing approaches to understanding the function of the system (the brain in its relation to body and environment) potentially allow for the mapping of dynamical relations among the embodied, experiential and affective, and intersubjective elements of the self-pattern in a way that is reflected in neural processes. Predictive processing suggests that these dynamical relations are hierarchically arranged, and this would be one way (although not the only way) to see that this explanation may also show how the very basic (embodied, experiential and affective) aspects of the self-pattern relate to more complex (cognitive, reflective, intersubjective and narrative) aspects.

Predictive processing accounts also suggest explanations of the failures of self-related experience found in psychopathologies. Seth (2013), for example, reviews research on deficits in predictive processing in psychiatric disorders affecting self-representation. Comparator models of disturbances in selfhood, for example, in schizophrenic delusions of control (Frith, 1992, 2011; Gallagher, 2000, 2004), foreshadow such predictive models of explanation. On predictive models, delusions may arise from the dominance of top-down predictions suppressing anomalous exteroceptive prediction errors (Fletcher and Frith, 2009). “This view has been finessed in terms of abnormal encoding of the relative precision of priors and sensory evidence to account for a broad range of psychotic symptoms” (Seth, 2013, p. 571). Seth thus proposes predictive processing explanations (especially involving interoceptive processing related to selfhood) for alexithymia (deficits in emotional awareness), psychosis and anxiety, disorders of body image and body ownership, depersonalization, and derealization.

## Conclusion

In response to skepticism about how the elements of a self-pattern are dynamically related to each other, such that they constitute a relatively coherent, if not strictly unified pattern, we have suggested three ways in which we can discover or investigate such dynamical relations. First, we suggested that a self-pattern is reflectively reiterated in its narrative component. Various components and dynamical relations of the self-pattern are reflected in self-narratives in a way that help us make sense of these relations. Second, they are revealed in the way that they break down in pathologies. The empirical and clinical exploration of psychiatric or neurological disorders can contribute to an understanding of the precise nature of the dynamical relations in a self-pattern. Third, as outlined in the previous section, predictive processing approaches to neuroscience can help explicate the dynamical relations that constitute the self-pattern as they are reflected in processes that bridge brain, body, and environment.

It should be clear from reviewing some of the evidence in support of these suggestions that explicating the dynamical relations that constitute a self-pattern is a complex task that requires an interdisciplinary approach. Neuroscientific predictive processing accounts constantly make reference to organism and environment, and can be enhanced by explications of embodied cognition, phenomenology, and psychopathology. Studies of psychopathology are enlightened not only by neuroscience, but also by clinical work and the analysis of patients’ self-narratives. The analysis of such narratives can deepen our understanding of the dynamical relations amongst different first- and second-person aspects of the self-pattern and reveal the complexities of human existence.

In this respect the phenomenological approach taken by de Haan et al. (2015), which integrates neuroscientific, pathological studies, and self-narrative (based on phenomenological interviews) is a good example of the kind of work that needs to be done to further the explication of a pattern theory of self and our understanding of the dynamics of the self-pattern. Their study of the experiences of OCD patients who have undergone Deep Brain Stimulation (DBS) treatment shows changes (1) in affordance structure (in terms of a patient’s pragmatic relations with world and others), (2) in affect (moods and feelings), and (3) in reflective, second-order evaluations about their interactions and themselves. They rightly argue that these neurological effects are “global and thorough” indicating “a pattern among these experiences … [where] all of these elements seem to play a role” (de Haan et al., 2013, p. 6). Admittedly, trying to explicate the holistic character of these kinds of experiences is, as they suggest, “tricky business”; nonetheless, the “focus on *dynamics*” is the way to start mapping out how various factors in a self-pattern relate to one another. Accordingly, it is only through such empirical, clinical, and phenomenological studies that we will be able to sort out how the dynamics of the self-pattern work, and how they may be different in pathological cases or become readjusted in cases of clinical treatments.

## Author Contributions

All authors listed have made a substantial, direct, and intellectual contribution to the work, and approved it for publication.

## Conflict of Interest Statement

The authors declare that the research was conducted in the absence of any commercial or financial relationships that could be construed as a potential conflict of interest.

## Footnotes

1 The idea of dynamical patterns with fields of prediction and control in the domain of psychology are prefigured in earlier research in theoretical biology (Rosen, 1970; Maturana and Varela, 1991) and cybernetics (McCulloch, 1965). Consistent with earlier research such as this, we take “dynamical pattern" to involve a non-linear, adaptive, anticipatory, feedback system comprising processes of interdependent elements which are determined not only by their properties and fields, but also by their relationships and functions within the whole.

2 To be clear, we’ll use the term “self-model” to refer to Metzinger’s concept, as shorthand for a set of self-related processes generated in the brain. In contrast, we’ll use “self-pattern” to signify a pattern that constitutively includes both neural and extra-neural factors and their dynamical relations.

3 The phrase “in-the-world” is found in the phenomenological philosophy of Heidegger and Merleau-Ponty. It signifies a structural characteristic of human existence rather than a geographical location. Its meaning relates, in part, to concepts such as “situation” in Dewey (1938), where the agent is not only always situated, but the situation is always defined relative to the agent; Gibson’s (1977) notion of “affordance”; or Koffka’s (2013) gestalt notion of “behavioral environment.”

4 In Daly and Gallagher (unpublished; also see Hutto and Gallagher, 2017) we argue that in the therapeutic context, narratives not only help us to understand patients’ experiences, but can also operate as a therapeutic tool that allows them to incorporate any new awareness or life adjustments into a continuing narrative. We can formalize the use of narrative in therapeutic contexts – not just in the sense of using a narrative therapy, but also in conjunction with other therapies.

5 Styron, 1990; Manning, 1994; Wurtzel, 1995; Foster Wallace, 1998; Wolpert, 1999; Solomon, 2001; Stirman and Pennebaker, 2001; Belle Doucet, 2003; O’Brien, 2004; Broome, 2005; Fuchs, 2005; Barton et al., 2008; Westerbeck and Mutsaers, 2008; Radden, 2009; Stein, 2012; Flint and Kendler, 2014; Opel et al., 2014; Ratcliffe, 2014; Trigg, 2016; Kelly, 2017a,b; Kirk et al., 2017; Stanghellini, 2017.

6 As one reviewer noted, for a full account of the relations that exist between neuronal processes and aspects of the self-pattern one would have to consider the different timescales involved and their non-linear dynamical relations. This is not something we can discuss here, but see Varela (1999) and Gallagher (2017).

7 The FEP aims to offer a unified brain theory that can account for the diverse empirical data related to action, perception, and learning. As Friston (2010) writes: “The FEP says that any self-organizing system that is at equilibrium with its environment must minimize its free energy. The principle is essentially a mathematical formulation of how adaptive systems (that is, biological agents like animals and brains) resist a natural tendency to disorder” (p. 127).

8 This may be one way to address a worry raised by Beni (2016, p. 3731), namely that it can be particularly difficult to account for the relation of the very different kinds of aspects of the self-pattern, across different dimensions, since “the dynamically constituted self-pattern is not woven around a central self that has its own independent existence.” This implies that one acknowledges the diversity of elements that enter into the self-pattern, and at the same time allow for the possibility that in any single organism there may be some degree of integration reflected at the level of neurobiology.

9 We take this basic idea of the FEP (if not the precise vocabulary used to describe it) to be consistent with enactivist approaches to cognition and the notion of system autonomy. “An autonomous system produces and sustains its own identity […] and thereby establishes a perspective from which interactions with the world acquire a normative status” (Thompson and Stapleton, 2009, p. 25). Here one could say much more about the relation between the FEP and the concept of autopoiesis (and its associated concept of autonomy) as found in enactivist accounts (see Bruineberg et al., 2016; Gallagher and Allen, 2016; Bitbol and Gallagher, 2018). Enactivists discount notions of hierarchical processing, and distinctions between top-down and bottom-up, or internal versus external, in favor of more gestalt-like relations (Gallagher, 2017). These are not issues we pursue here. The point we wish to make here is limited to the idea that predictive processing in general, i.e., disregarding important differences between internalist and enactivist conceptions of it, can provide a sense of how neuronal processing reflects dynamical relations in the self-pattern.

10 Apps and Tsakiris (2014, p. 91) note the influence of culturally shaped priors on predictive processing. “There is also evidence of more long-term contextual influences on self-recognition related priors, highlighted by the role that cultural and societal effects have on self-other decision-making. For instance, self-other face recognition has been shown to be different across cultures….”
